# Artificial intelligence predicts the progression of diabetic kidney disease using big data machine learning

**DOI:** 10.1038/s41598-019-48263-5

**Published:** 2019-08-14

**Authors:** Masaki Makino, Ryo Yoshimoto, Masaki Ono, Toshinari Itoko, Takayuki Katsuki, Akira Koseki, Michiharu Kudo, Kyoichi Haida, Jun Kuroda, Ryosuke Yanagiya, Eiichi Saitoh, Kiyotaka Hoshinaga, Yukio Yuzawa, Atsushi Suzuki

**Affiliations:** 1 Department of Endocrinology and Metabolism, Fujita Health University, Toyoake, Aichi Japan; 2 IBM Research, Tokyo, Japan; 3 Business Process Planning Department, The Dai-ichi Life Insurance Company, Limited, Tokyo, Japan; 4 IT Business Process Planning Department, The Dai-ichi Life Insurance Company, Limited, Tokyo, Japan; 5 Division of Medical Information Systems, Fujita Health University, Toyoake, Aichi Japan; 6 Department of Rehabilitation Medicine, Fujita Health University, Toyoake, Aichi Japan; 7 Department of Urology, Fujita Health University, Toyoake, Aichi Japan; 8 Department of Nephrology, Fujita Health University, Toyoake, Aichi Japan

**Keywords:** Type 2 diabetes, Diabetes complications

## Abstract

Artificial intelligence (AI) is expected to support clinical judgement in medicine. We constructed a new predictive model for diabetic kidney diseases (DKD) using AI, processing natural language and longitudinal data with big data machine learning, based on the electronic medical records (EMR) of 64,059 diabetes patients. AI extracted raw features from the previous 6 months as the reference period and selected 24 factors to find time series patterns relating to 6-month DKD aggravation, using a convolutional autoencoder. AI constructed the predictive model with 3,073 features, including time series data using logistic regression analysis. AI could predict DKD aggravation with 71% accuracy. Furthermore, the group with DKD aggravation had a significantly higher incidence of hemodialysis than the non-aggravation group, over 10 years (N = 2,900). The new predictive model by AI could detect progression of DKD and may contribute to more effective and accurate intervention to reduce hemodialysis.

## Introduction

Today, type 2 diabetes mellites (T2DM) is a worldwide burden afflicting developed and developing countries^1^. Chronic hyperglycemia and the subsequent accumulation of advanced glycation end-products result in multiple complications, including micro- and macrovascular diseases^2^. Among them, diabetic kidney disease (DKD), such as diabetic nephropathy, is the most frequent cause of hemodialysis (HD) and is associated with cardiovascular diseases^3^. Several clinical risk factors, such as hyperglycemia, dyslipidemia, hypertension and smoking, are related to the progression of DKD^4^. Microalbuminuria is known to be a good predictor of further progression of diabetic nephropathy and subsequent cardiovascular diseases^5^ and early intervention for DKD, such as anti-hypertensive medicine, could induce remission of DKD with microalbuminuria^6–9^. However, a more precise predictive model is needed for the very early intervention in DKD to prevent its further progression in diabetes patients without apparent symptoms or signs.

Artificial intelligence (AI) is changing our modern life and, in medicine, AI has two main branches, virtual and physical^10^. The physical branch includes robotics, which can assist surgery and rehabilitation. The virtual branch includes informatics, which is expected to assist physicians in their clinical diagnosis and treatment decisions. The recent progress of machine-learning, with big data analysis, is contributing greatly, especially in the field of clinical imaging^11,12^, pharmacokinetics^13^, genetics^14^ and oncology^15^. However, there is so far little information about predictive models of prognosis and/or progression of complications in life-style related diseases, such as T2DM^16–19^.

In general, clinical studies are designed to elucidate specific clinical risk factors by arranging background data or conditions before recruitment. On the other hand, we performed clinical medicine under non-arranged conditions. Therefore, population-based analysis is used for the assessment, considered as the so-called, real-world setting. However, the analyses have some disadvantages, such as many confounding factors which may cause several biases affecting the main conclusion. We hypothesized here that AI could provide more useful analysis by big-data-based machine learning without preconception.

In this study, we constructed a new predictive model of DKD in diabetes patients by big data machine learning, based on electronic medical records (EMR).

## Results

**Figure 1:**
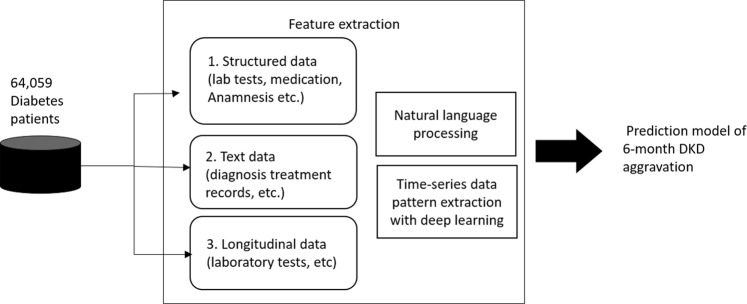
Feature extraction for deep learning. Clinical features for the predictive model of 6-months aggravation of diabetic kidney disease (DKD) were extracted using three different approaches: structural data, text data and longitudinal data from the electronic medical records (EMR) of 64,059 type 2 diabetes patients.

From 858,660 EMR, we extracted 451,584 cases with relevant clinical data. According to our criteria, 64,059 patients could be defined as T2DM. From these patients, we extracted the clinical features using three different approaches: structural data, text data and longitudinal data from EMR (Fig. 1).

**Figure 2:**
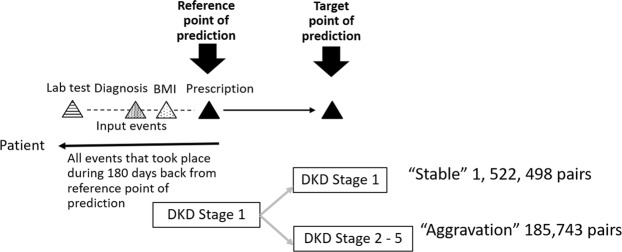
Extraction of “Stable” and “Aggravation” groups of diabetic kidney disease (DKD) for 6 months. We constructed 180 days-long event pairs between the reference point and target point of prediction in stage 1 DKD patients and obtained 1,708,241 pairs, including 1,522,498 in the stable group and 185,743 in the aggravation group. Then, we selected 15,422 in the stable group and 15,388 in the aggravation group by under-sampling.

During this process, AI extracted structural features such as laboratory tests, diagnosis, prescription and ICD 10 codes. AI picked up the past history, current diseases and prescriptions from the EMR text by natural language processing. Then, we constructed 180 days-long event pairs between the reference point and target point of prediction in stage 1 DKD patients and obtained 1,708,241 pairs, including 1,522,498 in the stable group and 185,743 in the aggravation group (Fig. 2). Finally, we selected 15,422 in the stable group and 15,388 in the aggravation group by under-sampling.

**Figure 3:**
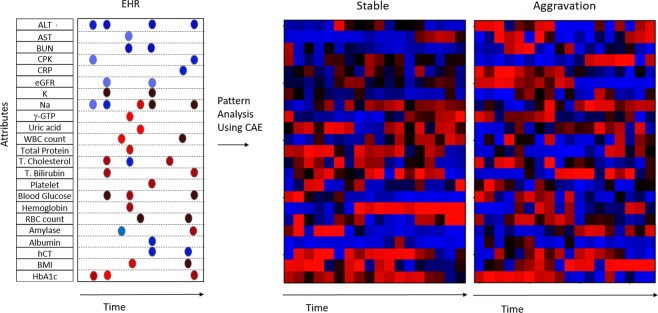
Time-series data pattern extraction with deep learning. Artificial intelligence extracted raw features during the 6 months prior to the reference point of prediction for selected 24 factors to reveal time series patterns relating to 6-month DKD aggravation, using a convolutional autoencoder (CAE) and inverse analysis. Red and blue mean high and low values, respectively, and brightness of the color mean the magnitude of the values.

At first, we examined how much the information that longitudinal data of EMR records have affected the DKD prediction. To this end, for those “Stable” and “Aggravation” groups, AI extracted raw features during the 6 months prior to the reference point of prediction for selected 24 factors to reveal typical time series patterns relating to 6-month DKD aggravation, using a convolutional autoencoder^20,21^ (Fig. 3). The 24 factors whose longitudinal information would affect the DKD were selected before the analysis. Figure 3 shows extracted typical time series patterns for “Aggravation” and “Stable” groups on the right, including some intriguing time series patters that creatine phosphokinase (CPK) and body mass index (BMI) have a conspicuous increasing patterns for the aggravation group. The results of above autoencoder experiment showed the importance of taking account of longitudinal data.

Second, AI also constructed the predictive model with 3,073 features, including longitudinal data using logistic regression analysis (Table 1). We used longitudinal explanation variables by summarizing past 180-day EMR records using average, standard deviation, and so on, taking the previous experiments into account. We then performed 5-fold cross validation and obtained a predictive evaluation result for each fold. The resultant average of the AUC was 0.743 and the average accuracy was 71%. Interestingly, 180-day statistical scores of laboratory tests before each reference time point seem to have good influence on prediction of DKD stage defined by urinary protein in 180 days. Therefore, the aggravation of urinary protein observation is strongly affected by its variance over the past 180 days. As actually shown in Table 2, as feature categories are added to the model, prediction performance improved. We observed that conspicuous improvement was shown when longitudinal features were added. Table 3 showed the resultant confusion matrix of our prediction.

**Table 1:** Extraction of time-series data and text data by natural language processing.

Category	Number of characteristics
**Structural data**	
Laboratory tests	168 (24 × 7)
Serum: Albumin, ALT, Amylase, AST, BG, BUN, CPK, CRP, eGFR, Creatinine, γ-GTP, HbA1c, Hemoglobin, Hematocrit, K, Na, Platelet, RBC, WBC, Total bilirubin, Total cholesterol, Total protein, Uric acid	Added to latest values, each feature has been extracted by longitudinal data series:
	1. Mean of all data
	2. The difference of the highest value and the lowest value
	3. S.D. of all data
	4. The difference of the last and first monthly means
	5. Mean of monthly mean data
Urine: Albuminuria, Protein	6. S.D. of monthly mean data
Profile	6
Prescription (YJ code)	408
ICD 10 (top 3 numbers)	1,265
**Text data from electronical medical records**	
Past history of diseases	613 (names of diseases)
Current disease	613 (names of diseases)

ALT: alanine aminotransferase, AST: aspartate aminotransferase, BG: blood glucose, BUN: blood urea nitrogen, CPK: creatinine phosphokinase, CRP: C-reactive protein, eGFR: estimated glomerular filtration rate, γ-GTP: γ-glutamyl transpeptidase, K: potassium, Na: sodium, RBC: red blood cell, WBC: white blood cell.

**Table 2:** Accuracy of prediction in each model.

Features	AUC	Accuracy
Profile	0.562	0.548
Profile + ICD10	0.562	0.557
Profile + ICD10 + YJCode	0.613	0.594
Profile + ICD10 + Blood Tests (latest)	0.644	0.606
Profile + ICD10 + YJCode +Blood Tests (latest and longitudinal)	0.656	0.610
Profile + ICD10 + YJCode +Blood Tests (latest and longitudinal) +Urinary Tests (latest and longitudinal)	0.729	0.691
Profile + ICD10 + YJCode +Blood Tests (latest and longitudinal) +Urinary Tests (latest and longitudinal) +Current Disease + Disease History	0.743	0.701

AUC: area under the curve; ICD10: 10^th^ revision of the International Statistical Classification of Diseases and Related Health Problems, YJ code: national health insurance drug list code.

**Table 3:** Confusion Matrix of Prediction Results.

	Predicted: Stable	Predicted: Aggravation
Actual: Stable	12,822	2,600
Actual: Aggravation	6,261	9,127

**Figure 4:**
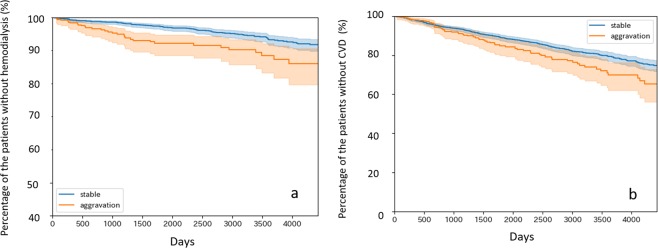
Kaplan-Meier survival analysis for hemodialysis (**a**) and cardiovascular disease (CVD) (**b**) after the first visit in the stable and aggravation groups. (**a**) The blue line shows the percentage of patients without hemodialysis in the “Stable” group (n = 2,477) at each time point, while the red line shows that of the “Aggravation” group (n = 423). Log rank test result marked P = 0.00024. (**b**) The blue line shows the percentage of patients without a cardiovascular event in the “Stable” group (n = 2,367) at each time point, while the red line shows that of the “Aggravation” group (n = 407). Log rank test result marked P = 0.01434. In this study, cardiovascular events are defined as hospitalized heart failure, myocardial infarction, performance of coronary artery bypass grafting, percutaneous coronary intervention, and death due to heart disease. The shaded areas show 95% confidence interval.

Third and last, we examined long-term relations with this 180-day prediction. When using the same “Stable” and “Aggregation” label for the patients, the DKD aggravation group had a significantly higher incidence of HD than the stable group over 10 years (Fig. 4a). Cardiovascular events were also more frequent in the DKD aggravation group than in the stable group (Fig. 4b).

## Discussion

In this study, we showed that AI could predict the progression of DKD using big data machine learning, according to the EMR of T2DM patients. Our study used three novel approaches to improve the predictive capacity of disease-specific complications. First, we constructed a new predictive model of diabetic complications before the patients showed clinical signs or symptoms such as microalbuminuria. Second, we used big EMR data for machine-learning by AI without any objective of clinical research; we included cases not defined clinically as T2DM in their text on EMR. Third, AI used time-series data from 6 months before the reference periods and predicted the progression of DKD for 6 months after the reference periods.

DKD is one of the most common diabetic complications and its progression results in hemodialysis for end-stage renal disease (ESRD)^3,22^. DKD is the major cause of hemodialysis in many countries^3^. Diabetes patients with normoalbuminuria have been reported to progress to microalbuminuria at 2.8%/year^23,24^. Because microalbuminuria is considered to be an early marker predicting diabetic nephropathy and subsequent ESRD, remission of microalbuminuria should mean less ESRD in the future^25,26^. There are several reports concerning the remission of early stage DKD, such as diabetic nephropathy at stage 2, where patients have 30–300 mg urinary microalbumin/day^6–9^. Theoretically, the earlier the intervention for DKD, the better the outcome we can expect in terms of remission. However, early intervention in stage 1 DKD must be less cost-effective and has the risk of overdiagnosis and/or overtreatment. Other biomarkers in diabetes patients with normoalbuminuria, such as urinary L-type fatty acid binding protein and serum tumor necrosis factor-α and its receptors, could also be surrogate markers of diabetic nephropathy^27^, but none of these markers is perfect. Liao *et al*. recently reported that urinary proteomics analysis could be useful to detect early diabetic nephropathy and that the haptoglobin-to-creatinine ratio might provide a better predictive value for early renal functional decline in 4.2 years than the microalbumin-to-creatinine ratio^28^. In this study, we constructed a model of early stage DKD at stage 1 to 2 diabetic nephropathy. With this approach, we could define T2DM at an early stage of DKD but with a higher future risk of its progress. It may be beneficial to provide more intensive care, such as statins and anti-hypertensive medicine, for these patients.

Proteinuria is related to atherosclerosis, resulting in cardiovascular diseases, such as ischemic heart disease and apoplexy^29^. For diabetes patients, we used microalbuminuria as a surrogate marker to predict ESRD and other atherogenic cardiovascular events^30^. We showed here that progression of DKD in 6 months could result in a higher incidence of hemodialysis due to chronic renal failure in our patients over 10 years. Furthermore, the unstable proteinuria group had a higher incidence of cardiovascular events than the stable non-proteinuria group. These results suggest that very early intervention to reduce proteinuria could contribute to a better prognosis for both renal and cardiac diseases. Many countries have progressed to super-aging societies and elderly patients are liable to have several diseases at the same time. Therefore, clinical medicine in super-aging societies is more complicated and clinical trials to find effective treatments are more difficult. In previous works to predict diabetes complications, they calculated the risks with several clinical information such as current age, sex, ethnicity, smoking status, presence or absence of microalbuminuria or worse, and laboratory data for diabetes, hypertension and dyslipidemia^31^. With this approach, we could pick up known risk factors according to the previous report but could not include unknown risk factors. In addition, when we perform the clinical trials to prove the efficacy of the treatment, we need to register many untreated patients as control. In our approach to find an AI-supported predictive model for chronic diseases such as T2DM, we can elucidate the combination of clinical risk factors with less expense and less effort than current clinical trials. AI has the advantages of improving clinical medicine in the field with digital data such as imaging^11,12^, pharmacokinetics^13^, genetics^14^ and oncology^15^. Recent studies with AI in the field of diabetes represent a diverse and complex set of innovative approaches that aim to transform diabetes care in four main areas: automated retinal screening, clinical decision support, predictive population risk stratification, and patient self-management tools^32^. AI could improve imaging techniques such as diabetic retinopathy screening^33^, because digital imaging is an aggregation of many pixels with the same processing condition. Recently, the US Food and Drug Administration (FDA) permitted the marketing of the first medical device to use AI to detect diabetic retinopathy. There are several innovative studies using a machine-learning approach to develop phenotyping frameworks to detect diabetes^16^, the progression of diabetes^18^ and hypoglycemia^17^. However, an AI-oriented predictive model of diabetic complications has not yet been developed. Our current study suggests that AI could support our decision to reduce future clinical events at an early stage of complications in chronic diseases such as T2DM.

There are some limitations to this study. First, the information obtained from each EMR, especially from the medical doctors’ records, varies considerably and we could not unify the data extraction from each patient. Second, the duration between each laboratory test was not uniform and depended on the individual patient. Third, this study was carried out in a single center and has not been reevaluated using EMR from other institutions. Fourth, we could not find any relationship between progression of DKD for 6 months and medication. Because the patients without DKD are likely to be treated less intensively, we suggest that the medication itself may not have affected the progression of DKD in this study. Therefore, we still need prospective study to prove very early intervention to DKD could prevent macrovascular events including ESRD and CVD in T2DM patients.

In conclusion, the new predictive model using AI could detect the progression of DKD, which may contribute to more effective and accurate intervention to reduce hemodialysis and cardiovascular events.

## Methods

### Definition of T2DM and the EMR used for this study

We started to use the EMR system in our hospital in 2005 and had 858,660 EMR in 2016. There are 407,076 EMR without any clinical data, so we could extract 451,584 EMR in total. Among them, we found 64,059 patients with a diagnosis of T2DM according to our criteria as follows: (1) T2DM was recorded in the medical billing, (2) the HbA1c level was equal to or above 6.5% (NGSP), (3) the fasting plasma glucose level was equal to or above 126 mg/dL, except for in an emergency room, (4) the postprandial plasma glucose level was equal to or above 200 mg/dL, except for in an emergency room, (5) anti-diabetic medicine (137 drugs) was prescribed. This study was approved by the Fujita Health University Ethical Committee (HM17-159). Informed consent from each patient was not available; therefore, the opportunity to opt out of this research was announced on our homepage (http://www.fujita-hu.ac.jp/~endylabo/research/watson_01/index.html). All private personal information was protected and removed during the process of analysis and publication. All data associated with this study are present in the paper or in the Supplementary Materials.

### Staging categorization of DKD in this study

Diabetic nephropathy is defined by albuminuria and a decrease of the estimated glomerular filtration rate (eGFR). In typical diabetic nephropathy, the microalbuminuria precedes the decrease of the eGFR because hyperglycemia induces glomerular damage resulting in a dysfunction of the barrier system in glomerulus. However, patients with low eGFR without apparent proteinuria have been increasing in number recently because of the treatment and the aging population. Therefore, we have recently started to use DKD instead of diabetic nephropathy^3^. In this study, we defined the DKD stage by proteinuria, the albumin to creatinine ratio or the eGFR at stages 1 to 4 (Table S1). Stage 5 is defined as maintenance hemodialysis or continuous ambulatory peritoneal hemodialysis.

### Predictive model of DKD

Focusing on DKD, one of the diabetic complications that will incur heavy treatment, including dialysis, if aggravated, a model was developed to predict its progression. Based on laboratory test results, this model uses the stage of DKD (one to five, Table S1) as labels and predicts whether the diabetes of DKD stage 1 patients will progress, in terms of DKD stage, after 180 days. A predictive model was created using a variety of features, including patient profiles, name of the disease and treatment (details of treatment and medication), extracted from EMR. These records are in the clinical data base system of Fujita Health University Hospital.

### Label definition

**Figure 5:**
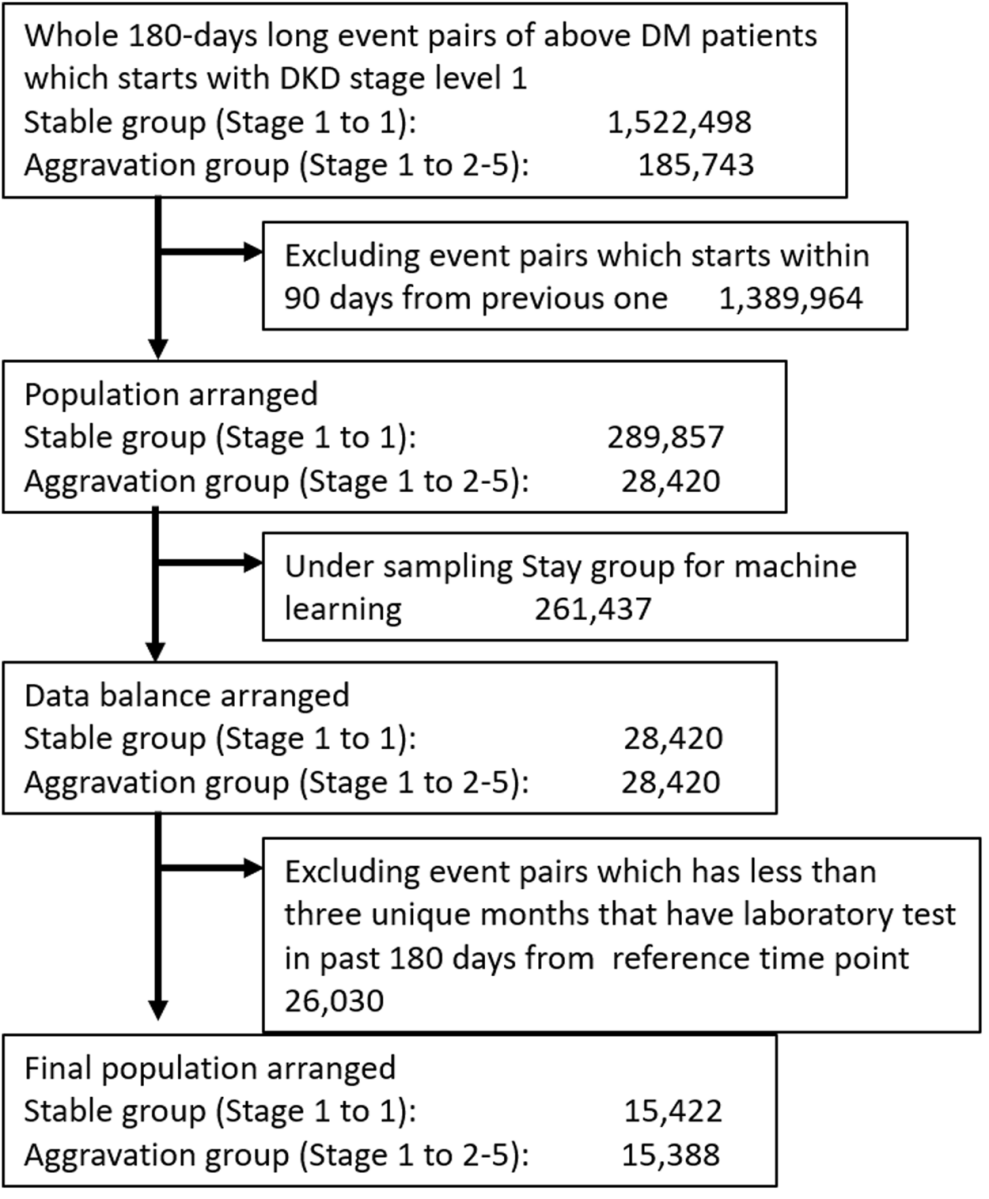
Study population. After identifying labeled pairs, we manually under-sampled the pairs to balance the numbers. In this test, the stable group was much larger than the aggravation group, so pairs in the stable group were under-sampled to match the numbers better. We used these labeled pairs for supervised learning; the final numbers are 15,422 for the stable group and 15,388 for the aggravation group. The characteristics of the study population are shown in Table S2.

Labels for prediction are created from the DKD stage change in 180 days. We first find any pair of events of the “reference point” and “target point”, which are measured around 180 days apart. Closely measured pairs were filtered to avoid using similar data. Then, we labeled the pairs as the stable and aggravation groups. The stable group (n = 1,522,498) were in stage 1 at the reference and target points (Fig. 2). The aggravation group (n = 185,743) are those whose DKD stage at the reference point was 1 but who progressed to stage 2 or more at the target point. We excluded 1,389,964 event pairs whose reference and target points were less than 90 days apart. After this exclusion, there were 289,857 patients in the stable group and 28,420 in the aggravation group. In order to adjust the numbers of each group for machine learning, we performed under-sampling of the stable group. Finally, we excluded 26,030 event pairs which had less than three unique months that had had laboratory tests in past 180 days from reference point. The final population for this study was selected as 15,422 in the stable group and 15,388 in the aggravation group (Fig. 5).

### Structured features

We extracted a variety of features from the EMR. They include laboratory tests, profiles, medication, disease history and so on. We used the values which were collected at most 180 days prior to the reference point. Table 1 shows the categories of structured features and their numbers. Among these feature values, we created processed features by statistical aggregation of feature sequences, because we have a time-series of measured values for each feature. We calculated 180-day S.D., mean, and others described in Table 1 and then used these as the features.

### Unstructured features and text processing

Other than structured features, we processed texts from medical examinations and nutrition consultations. The texts are recorded in the free text format for each consultation. From those we extracted disease names of current and historical diseases as keywords by traditional natural language processing. We extracted those keywords using disease name dictionaries for name aggregation. Other than keywords extraction, we also used topic information as features. As using the medical text records as a corpus, we conducted topic analysis using Latent Dirichlet Allocation (LDA)^34^, which are commonly used for topic extraction. Such extracted topics are also used for our features.

### Time series pattern analysis

To selected 24 factors whose longitudinal information would affect the DKD, we conducted an analysis to find time series patterns relating to 6-month DKD aggravation using a convolutional autoencoder^20,21^. In the convolutional autoencoder for the input vector X of time series data of 24 factors, the encoder has 5 hidden layers consisting of: 1) one-dimensional convolutional layer with 64 24 × 3 filters for X; 2) a max-pooling layer with 1 × 2 filter; 3) a one-dimensional convolutional layer with 64 1 × 3 filters per map; 4) a max-pooling layer of 1 × 2 filter; 5) a fully connected layer which extracts a hidden representation as 128 neurons as function f(X), while the decoder is the transposed convolution^35^, which constructs a reverse function g(.) where X = g(f(X)). We then minimize the reconstruction error of X − g(f(X)) to learn the autoencoder. Next we learn the classification model to predict “Aggravation” or “Stable” using the extracted hidden representation. From those results, we generated the typical input time series patterns by inverse analysis, which finds maximum input time series values so that the hidden representation corresponding to “Aggravation” or “Stable” is activated. Details of the network architecture and mathematical definitions are described in our previous paper^21^.

### Prediction model

**Figure 6:**
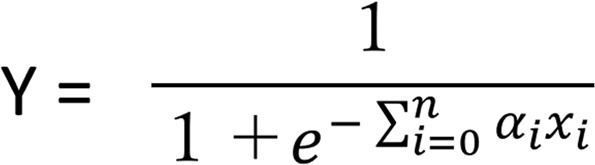
Formula to predict the probability of the DKD stage a half-year later. The probability of the DKD stage a half-year later as Y, where x_i_ are the input variables (the number is n) and α_i_ are parameters, which were calculated using logistic regression.

We applied logistic regression using the Python code with scikit-learn library (https://scikit-learn.org/) for model solving. Among many machine learning packages including R (https://www.r-project.org/), SPSS (https://www.ibm.com/analytics/spss-statistics-software), Matlab (https://www.mathworks.com/products/matlab.html), SAS (https://www.sas.com/home.html), Weka (https://www.cs.waikato.ac.nz/ml/weka/) and other, we chose scikit-learn because our feature extraction processes are written in Python. Due to the large number of explanation variables, we used L2-reguralization to avoid overfitting. With regularization, we also used a stepwise method to choose explanation variables. To speed up the computation, we adopted a concurrent algorithm to choose those variables. Using the feature set once selected, we used a 5-fold cross validation method to evaluate the prediction performance. Figure 6 shows the formula used to predict the probability of the DKD stage a half-year later.

### Long-term effect of DKD aggravation

In predicting 180-day DKD stage aggravation, it is interesting to know whether such short-term stage changes are related to changes in long-term patient status; the period is sufficiently long to observe even severe events, such as dialysis and other critical end points. To elucidate this short-term to long-term relationship, we conducted survival analysis using Kaplan-Meier methods, using two groups of 180-day DKD stage changes, namely the stable and aggravation groups. We estimated two Kaplan-Meier curves for the diabetic hard end points of hemodialysis and cardiovascular disease events. The cardiovascular disease events include hospitalized heart failure, myocardial infarction, coronary artery bypass grafting, percutaneous coronary intervention, and death from heart disease.

Observing the 180-day DKD stage change and to analyze each patient once, we collected 180-day DKD stage change data from the first visits of the patients to the hospital. More precisely, we collected the first DKD stage using laboratory test results from the patient’s first visit to the hospital and the second DKD stage using laboratory test results, which were taken between 180 and 240 days after the first visit. We then separated the data for the stable and aggravation groups. The definition of stable and aggravation is as we described for the predictive model. Finally, we estimated Kaplan-Meier curves from 240 days after the first visit to the last recorded date from the patient’s EMR. Note that, for the aggravation group, we excluded patients who already suffered from the hard end-points. This setting provides a fair and conservative investigation of the short-term to long-term relationship.

For survival analysis for hemodialysis, the number of patients in the stable group was 2,477; they remain at DKD stage 1. The number of patients in the aggravation group was 423, all of whom survived and at the DKD stage of 2 to 5 about 180 days after the first visit. For survival analysis for cardiovascular disease, the respective numbers are 2,367 and 407.

### Statistical analysis

To determine the relationship between half-year prediction and long-term tendency, we also conducted a survival analysis. We estimated Kaplan-Meier functions and the curves of occurrence of hemodialysis and cardiovascular diseases and carried out a Log-rank test. We prepared two groups of “Stable” and “Aggravation”, as described above, and applied Kaplan-Meier estimations using LIFELINES (https://lifelines.readthedocs.io/en/latest/), a python library of survival analyses. P < 0.05 was considered as statistically significant.

## Supplementary information

Dataset 1
