# Supplementary material for: Artificial intelligence predicts the progression of diabetic kidney disease using big data machine learning
Source: Sci Rep. 2019 Aug 14;9:11862. doi: 10.1038/s41598-019-48263-5 (PMC6694113; doi:10.1038/s41598-019-48263-5)
Supplement: Supplementary file 1 — Dataset 1 [file 41598_2019_48263_MOESM1_ESM.docx]

**Supplementary materials**

**Title: Artificial intelligence predicts the progression of diabetic kidney disease using big data machine learning**

**Authors:** Masaki Makino, Ryo Yoshimoto, Masaki Ono, Toshinari Itoko, Takayuki Katsuki, Akira Koseki, Michiharu Kudo, Kyoichi Haida, Jun Kuroda, Ryosuke Yanagiya, Eiichi Saitoh, Kiyotaka Hoshinaga, Yukio Yuzawa, Atsushi Suzuki

| **Stages** |  | |
| --- | --- | --- |
| Stage 1 | ACR < 30 mg/g・Cre  or  urine protein (-) | eGFR equal to or above  30 mL/min/1.73m^2^ |
| Stage 2 | ACR 30 -299 mg/g・Cre  or  urine protein (+/-)(+) |  |
| Stage 3 | ACR equal to or above  300 mg/g・Cre  or  urine protein (2+)(3+)(4+) |  |
| Stage 4 |  | eGFR <30 mL/min/1.73m^2^  Except for the patients on hemodialysis |
| Stage 5 |  | Patients on hemodialysis |

Table S1. Staging categorization of diabetic kidney disease

ACR:　albumin creatinine ratio, Cre: creatinine, eGFR: estimated glomerular filtration rate

| Index | Stable (N=15442) | Aggravation (N=15388) |
| --- | --- | --- |
| Gender (Male/Female) | 7771/7651 | 8547/6841 |
| Age (year) | 61.9±16.0 | 63.9±16.2 |
| HbA1c (%) | 6.29±0.19 | 6.66±0.24 |
| Creatinine (mg/dL) | 0.74±0.05 | 0.85±0.07 |
| Past history of cardiovascular diseases | 548 | 952 |

Table S2. Characteristics of the study population

| Loop1 | Albuminuria (Mean) | Uric acid (Standard deviation) | Creatinine (Difference  of highest and lowest values) | ICD10 top 3: M79 Other soft tissue disorder | Prescription of aminobenzoic acid alkaline ester |
| --- | --- | --- | --- | --- | --- |
| Loop2 | Uric acid (Standard deviation) | Albuminuria (Mean) | ICD10 top 3: E04 Other nontoxic goiter | Creatinine (Difference of highest and lowest values) | ICD10 top 3: M79 Other soft tissue disorder |
| Loop 3 | Uric acid (Standard deviation) | Albuminuria (Mean) | Creatinine (Difference of highest and lowest values) | ICD10 top 3: M79 Other soft tissue disorder | ICD10 top 3: E07 Other thyroid disorder |
| Loop 4 | Albuminuria (Mean) | Uric acid (Standard deviation) | Creatinine (Difference of highest and lowest val.) | ICD10 top 3: M79 Other soft tissue disorder | Creatinine (Mean) |
| Loop 5 | Albuminuria (Mean) | Uric acid (Standard deviation) | Creatinine (Difference of highest and lowest values) | ICD10 top 3: M79 Other soft tissue disorder | Creatinine (Mean) |

Table S3. Top 5 Features of 5-fold cross validation loops
